# Enhancing Patient Education in Multiple Myeloma: Cognitive Load and Socio-Emotional Adaptation

**DOI:** 10.1093/geroni/igaf122.758

**Published:** 2025-12-31

**Authors:** Sean Halpin

**Affiliations:** RTI International, Decatur, Georgia, United States

## Abstract

This study expands on Socio-Emotional Adaptation (SEA) Theory by integrating cognitive load concepts to examine the educational experiences of older adults with multiple myeloma (MM) undergoing autologous stem cell transplantation (ASCT). SEA Theory highlights how perceived control, adaptation, and relationships with caregivers modulate emotional responses to illness. Adding cognitive load underscores how these factors impact patients’ bandwidth to process health information. Over 150 hours of ethnographic observation of nurse-patient education sessions (N = 70), supplemented by patient interviews (N = 35) and clinician interviews (N = 7), were conducted over 18 months. Findings reveal barriers, including anxiety, overwhelming information, and lack of medical expertise, alongside facilitators such as tailored education and supportive social networks. By iteratively developing video-based tools addressing these barriers, the intervention decreased cognitive overload while enhancing emotional readiness. This case underscores how cognitive and socio-emotional models inform patient-centered educational practices for older adults with life-limiting illnesses.

